# Financial Strain and Health Status Among European Workers: Gender and Welfare State Inequalities

**DOI:** 10.3389/fpubh.2021.616191

**Published:** 2021-05-20

**Authors:** Lucía Artazcoz, Imma Cortès-Franch, Vicenta Escribà-Agüir, Fernando G. Benavides

**Affiliations:** ^1^Public Health Observatory, Agència de Salut Pública de Barcelona, Barcelona, Spain; ^2^Ciberesp ISCIII, CIBER en Epidemiología y Salud Pública (CIBERESP), Madrid, Spain; ^3^Department of Experimental and Health Sciences, Pompeu Fabra University, Barcelona, Spain; ^4^Institute for Research in Biomedicine, IIB-Sant Pau, Barcelona, Spain; ^5^Department of Pediatrics, Obstetrics and Gynecology, and of Preventive Medicine, Faculty of Medicine, Autonomous University of Barcelona, Barcelona, Spain; ^6^Health Inequalities Area, Centre for Public Health Research, Valencia, Spain

**Keywords:** financial strain, health', economic hardship, socioeconomic factors, gender

## Abstract

**Objectives:** Although in-work poverty has been increasing, in Europe policy about poverty and social exclusion tends to focus on labor market participation, independently of the level of remuneration and the quality of work, and studies about financial strain among workers, as well as on its relationship with health status, are still scarce. The objectives of this study were: (1) to compare the prevalence of financial strain among workers among different welfare state typologies, and (2) to examine whether the relationship between financial strain and health status differs by welfare state regime. For both objectives we examined whether there were gender differences.

**Methods:** We conducted a cross-sectional study using data from the 6th European Working Conditions Survey of 2015 and selected a subsample of all employees from the EU28 aged 16–64 years (13,156 men and 13,225 women).

**Results:** There were large differences in the prevalence of financial strain between welfare state typologies, which were not explained by individual factors. Additionally, differences across welfare regimes were greater among women. Nordic countries had the lowest prevalence (12.1% among men and 12.3% among women) whereas Southern European countries had the highest (49.5% among men and 47.9% among women). In both sexes and in all welfare state typologies, financial strain was associated with poor self-perceived health status and poor psychological well-being. Whereas, Southern European countries had the highest prevalence of financial strain, the magnitude of the association with health status was smaller than in other country typologies.

**Conclusion:** In Europe, policies are needed to address the specific structural factors leading to financial strain as well as its relationship with health status among workers.

## Introduction

Since the global economic crisis that started in 2007/2008, in-work poverty has been increasing (1). However, despite this trend, in Europe there has been relatively little policy debate and research about this topic (2), where policy about poverty and social exclusion tends to focus on labor market participation, independently of the level of remuneration and the quality of work (3).

The EU Statistics on Income and Living Conditions (EU-SILC) considers individuals to be at risk of in-work poverty if they work for over half of the year and have an equivalized yearly disposable household income below 60% of the median national household income (4). However, several studies indicate that objective measures of income do not capture the meaning of income adequacy to individuals, with people on low incomes not always reporting financial strain, which indicates that these two measures are different (5, 6). The concept of financial strain allows to understand the experience and meaning of poverty by examining the extent to which families meet their basic needs (7).

### Financial Strain and Health Status

Financial strain is one of the greatest stressors encountered during adulthood, and has been shown to be related to poor health status, independently of other measures of socio-economic position and demographic characteristics (8, 9). It can be linked to health because of inability to manage on the income which involves stress, anxiety, and helplessness (10, 11). It has also been reported that people with financial strain are more likely to have unhealthy behaviors (12).

Most studies about financial strain and health status have focused on elderly people whereas studies about the working population are scarce (13). Moreover, low personal wages, that are often the focus of studies on the working poor, are only one cause of financial strain, which depends on a household's overall resources and needs, and includes individual factors such as age, immigration status, employment and household characteristics (14). Additionally, due to the gender division of labor both the prevalence of financial strain and the relationship between financial strain and health status can differ by gender. For example, a study about the impact of job loss and job recovery reported that for men, employment recovery was insufficient to alleviate financial strain and associated health consequences, whereas in women regaining employment lead to health recovery (15).

### Role of the Welfare State Regimes

The prevalence of financial strain varies markedly between European countries (16) which can be explained, not only by differences in the individual factors associated with financial strain, but also by contextual factors, primarily associated with the levels of decommodification and defamilisation, which are closely related to welfare state typologies (14, 17).

Decommodification refers to “the degree to which social policies permit people to make and maintain their living at a socially acceptable level independent of market forces, without having to sell their labor power on the labor market” (18). In trying to explain country-specific differences in financial strain among workers it is important to consider the level of earnings as well as the level and availability of other sources of income, particularly transfers, which typically include old-age pensions, sickness leave, and unemployment benefits. Welfare states are related to labor market regulations which influence the level of unemployment as well as precariousness and, consequently, wages and the risk of financial strain. It is also important to consider in-work benefit programs with social transfers provided by public bodies to those living in poverty or in risk of falling into poverty which can increase the incomes of households that would otherwise be below the poverty threshold. Furthermore, there are non-contributory benefits like family allowances which also contribute to household income. Generally, higher levels of decommodification are correlated with lower levels of financial strain (19).

Defamilisation refers to the fact that welfare states, not only influence the degree of an individual's dependence on markets, but also a person's dependence on his or her own family. This concept is concerned with women's dependence on the family due to care obligations and a male breadwinner as well as the dependence status of young adults due to difficulties in labor market access and no access to transfers in their own right, and on the dependence of the elderly due to a lack of care options outside the family (20). Welfare state typologies with different defamilisation levels differ in women's participation in the labor markets and intergenerational dependencies. Examples of defamilisation instruments include free public childcare services, child benefit paid to families to cover some of the children's material needs, and parental leave to care for older or sick family members (17). Welfare state measures which promote female employment are expected to reduce in-work poverty. Intergenerational dependency can have an ambiguous effect: while for young workers, living with their parents will lower their risk of financial difficulties, the financial strain for the working parents will rise. However, the pooling of resources protects family members from being poor.

### Welfare State Regimes in Europe

Grouping EU countries according to their levels of decommodification and defamilisation yields five country groups: Nordic, Anglo-Saxon, Continental, Southern, and Eastern European countries (21). Nordic countries are characterized by high degrees of both decommodification and defamilisation. Macro and microeconomic policies stimulate maximum employment participation on a basis of gender equality. Anglo-Saxon countries have the lowest levels of decommodification of the five welfare state typologies, and low levels of defamilisation, with family care considered to be a private responsibility. The lack of public childcare creates a major barrier to female participation in the labor force. On the other hand, the Anglo-Saxon countries have gone the furthest in implementing in-work benefits (primarily tax credits) aimed at households with low earnings, specially single-parent households (22). Continental European countries have considerable levels of decommodification and—although less so—defamilisation, with family policy models characterized by high levels of traditional family support from the state, and low levels of support for female participation in the labor force. Several Continental countries devote considerable resources to family care provisions and active labor market measures (19). Southern European countries are characterized by low levels of decommodification and of defamilisation with low spending on family policies and lack of affordable childcare services. Family and inter-generational solidarity play a strong role, not only reducing work/care conflicts but also in providing protection against social risks (21). Finally, Eastern European countries have low levels of decommodification, and although women's labor market participation is high in most cases, there has been a process of re-familisation in the post-communist era. Southern and Eastern European countries have the highest level of precarious employment in the European Union (23).

Welfare state typologies can also determine the relationship between financial strain and health status due to “health resilience” by which some areas exhibit better health outcomes than would be expected given their level of deprivation. In these areas, health advantage is conferred by a complex range of factors such as health-related behaviors, social, religious, or ethnic support (11).

In summary, although poverty is one of the core objectives of the European Union's social inclusion strategy (24), policies are primarily focused on promoting labor force participation and knowledge about financial strain and its relationship with health status among workers is still scarce. The objectives of this study were: (1) to compare the prevalence of financial strain among workers among different welfare state typologies, and (2) to examine whether the relationship between financial strain and health status differs by welfare state regime. For both objectives we examined whether there were gender differences.

## Methods

### Study Design and Population

We conducted a cross-sectional study using data from the 6th European Working Conditions Survey (EWCS) of 2015. Details of the survey are reported elsewhere (25). For the purposes of this study, we selected a subsample of all employees from the EU28 aged 16–64 years. We excluded self-employed workers because their employment and working conditions are substantially different to those of salaried workers (26). Since health problems may reduce the chance of having a good quality job, to avoid possible reverse causation, we excluded from our analysis all individuals who reported a limiting long-standing illness (LLI). The presence of LLI was assessed through two questions: Do you have any illness or health problem which has lasted, or is expected to last, for more than 6 months? This was a dichotomous question and those who answered “yes” were further asked: “Are your daily activities limited because of this illness or health problem? Answers fell into three categories: workers who answered “yes, severely limited”, and “yes, somewhat limited were considered to have LLI (*n* = 2,693). The final sample for analysis included 13,156 men and 13,225 women.

### Variables

Although many studies about material deprivation have focused on specific indicators of material hardship such as food insecurity, energy poverty or housing security, for example (7), a single indicator of households' ability to make ends meet provides a valid subjective measure of overall financial strain (27, 28). Moreover, previous studies have shown that this indicator is closely related to material deprivation (29, 30). Therefore, financial strain was assessed via the question “A household may have different sources of income and more than one household member may contribute to it. Thinking of your household's total monthly income, is your household able to make ends meet…?”; answers fell into six categories, from “very easily” to “with great difficulty.” The variable was dichotomized, and individuals who answered “with some difficulty,” “with difficulty,” and “with great difficulty” were considered to suffer from financial strain.

### Health Outcomes

Data on self-perceived health status were collected by asking respondents to describe their general health as “very good,” “good,” “fair,” “poor,” or “very poor” (31). This variable was dichotomized by combining “fair,” “poor,” and “very poor” to indicate poor self-perceived health, and “very good” and “good” to indicate good perceived health. Subjective well-being was measured using the 5-item World Health Organization Well-Being Index (WHO-5) which is among the best and more used questionnaires assessing subjective psychological well-being (32). We created a dichotomous variable, where a score of ≤50 indicated poor psychological well-being, though not necessarily depression for which the usual score is ≤28 (33). This approach has been used in many previous studies based on working conditions surveys (34, 35). It should be noted that the prevalence of depression among people currently working is very low due to the “healthy worker effect.”

### Welfare State Typologies

Countries were grouped into five categories (21): Continental (Austria, Belgium, Germany, France, the Netherlands, and Luxembourg), Anglo-Saxon (Ireland and the United Kingdom), Eastern European (Czech Republic, Estonia, Hungary, Lithuania, Latvia, Poland, Croatia, Romania, Bulgaria, Slovenia, and Slovakia), Southern European (Cyprus, Greece, Spain, Italy, Malta, and Portugal) and Nordic countries (Denmark, Finland, and Sweden).

### Adjusting Variables

The analyses were adjusted for sociodemographic, employment and household characteristics. Participants were grouped into three age categories, 16–24, 25–44, and 45–64 years, and immigration status was determined by country of birth, with two categories, immigrants, and natives. Job category, which is a proxy for job qualification and socioeconomic status (36), was coded according to the 2008 International Standard Classification of Occupations (37), and grouped into three categories: upper (1 and 2), middle (3 to 5), and lower (6 to 9). Type of contract had four categories: permanent, fixed-term temporary, no contract, and other. Data on workers paid working hours were collected via the question: “How many hours do you usually work per week in your main paid job?” The responses for each question were summed and grouped into four categories: <30 h [part-time employment (PTE)], 30–40 h (reference category), and >40 h a week [long working hours (LWH)]. We assessed household characteristics in terms of cohabitation with a partner, with parents, with other workers at home, and with unemployed persons at home, all of which were dichotomous variables, as well as the number of children (0, 1, or >1).

### Data Analysis

First, we conducted a bivariate analysis using the chi-square test to test for gender differences in all dependent and independent variables, stratifying by welfare state typology. Second, to compare the prevalence of financial strain between welfare state typologies, we fit logistic regression models, unadjusted and adjusted for sociodemographic factors, employment, and household characteristics. Finally, to test for association between health outcomes and financial strain, we fit multiple logistic regression models adjusted for sociodemographic factors, employment, and household characteristics. Logistic regression models were stratified by gender and welfare state typologies and included weights derived from the complex sample design.

## Results

### Description of the Sample

Table 1 shows the general characteristics of the sample. Almost half of workers from Eastern and Southern European countries reported financial strain, whereas the lowest prevalence was observed in Nordic countries. In Continental, Anglo-Saxon, and Eastern European countries, financial strain was significantly higher among women. Eastern and Southern European countries had the highest prevalence of poor self-perceived health status, with no gender differences; in contrast, in Anglo-Saxon and Nordic countries the prevalence was lower among women. The prevalence of poor psychological well-being was higher in Anglo-Saxon countries.

**Table 1:** General description of the population by sex and welfare state typology.

	**Continental**		**Anglo-Saxon**		**Eastern Europe**		**Southern Europe**		**Nordic**	
	**Men *N* = 5,142**	**Women *N* = 5,109**	**Men *N* = 1,995**	**Women *N* = 1,919**	**Men *N* = 2,592**	**Women *N* = 2,871**	**Men *N* = 2,800**	**Women *N* = 2,746**	**Men *N* = 627**	**Women *N* = 580**
Financial strain	27.6	31.0^c^	17.8	24.4^c^	41.4	44.3^a^	49.5	47.9	12.1	12.3
Poor self-perceived health	12.7	12.9	12.9	8.7^c^	18.1	18.2	19.7	20.3	14.0	9.1^b^
Poor psychological well-being	10.5	14.4^a^	18.9	22.8^b^	14.2	15.2	11.9	14.0^a^	12.0	14.0
**Sociodemographic characteristics**										
***Age***										
16–24	10.5	9.5	14.0	12.6	8.4	6.7^a^	5.9	5.9	9.8	9.4
25–44	47.0	49.0	47.7	47.7	51.9	55.5	51.2	51.2	48.4	44.6
45–64	42.5	41.5	38.3	39.7	39.7	37.8	42.9	42.9	41.8	46.0
*Immigrant*	8.8	9.5	17.7	17.4	1.4	1.4	8.0	8.2	9.4	10.0
**Employment characteristics**										
***Job category***										
Upper	20.7	20.7^c^	34.7	36.2^c^	16.7	28.0^c^	17.0	22.9^c^	29.9	37.9^c^
Middle	38.6	62.4	33.1	53.7	30.8	50.3	39.8	56.6	38.3	52.1
Lower	40.7	16.9	32.2	10.1	52.5	21.7	43.2	20.5	31.7	10.0
***Working time***										
<30 h	9.1	38.8^c^	10.7	38.4^c^	7.4	13.2^c^	11.8	31.6^c^	10.4	15.3^c^
30–40 h (standard)	71.9	52.8	51.5	47.6	57.9	65.9	64.7	58.5	64.3	71.8
>40 h	19.0	8.4	37.8	14.0	34.7	20.9	23.5	9.9	25.3	12.9
***Type of contract***										
Permanent	84.6	81.2^c^	85.6	85.9	78.4	78.1^c^	74.4	68.6^c^	86.1	82.4
Fixed-term temporary	9.7	12.5	5.0	5.0	14.0	14.5	17.1	18.5	9.9	13.1
No contract	1.6	2.4	4.6	4.6	4.7	3.0	6.0	9.2	1.6	1.2
Other	4.1	3.8	4.8	4.8	2.9	4.3	2.5	3.7	2.4	3.3
**Household characteristics**										
*Living with a partner*	70.3	67.2^b^	67.3	67.1	66.2	70.3^b^	65.9	66.7	66.8	61.6
*Living with parents*	8.6	7.4	16.3	12.0	25.3	17.6	20.0	16.9	5.9	5.3
***Number of children***										
0	57.6	48.2^c^	59.7	53.7^b^	60.3	47.0^c^	54.4	45.7^c^	54.9	54.0
1	16.9	22.7	16.0	18.5	20.7	28.2	23.1	25.9	16.1	17.2
≥2	25.5	29.1	24.3	27.8	19.1	24.8	22.4	28.4	29.0	28.8
*Other workers at home*	62.8	67.4^c^	68.0	74.9^c^	61.8	70.6^c^	61.7	59.1^c^	58.9	58.3
*Unemployed at home*	6.3	6.0	8.0	8.5	12.4	8.7^c^	18.9	14.1^c^	5.9	6.0

*Employees aged 16–64 years (%). P-values compare men and women in each country typology. 6th European Working Conditions Survey, 2015*.

a *P < 0.05,*

b *P < 0.01,*

c *P < 0.001*.

The proportion of young employees was higher in Anglo-Saxon countries and lower in Southern Europe; the proportion of immigrants was larger in Anglo-Saxon countries but was <2% in Eastern European countries.

Regarding employment characteristics, in all country typologies, men were more likely to work in the lower occupational category and to work long hours, whereas women were more likely to work part-time. In both sexes, fixed-term temporary contracts were more common in Southern and Eastern European countries, whereas working without a contract was more common in these latter typologies and in Anglo-Saxon countries.

With regard to household characteristics, people from Eastern and Southern European countries were more likely to live with parents. Living with unemployed persons at home was more common among workers from Southern European countries.

### Prevalence of Financial Strain

Table 2 shows differences in the prevalence of financial strain by gender and welfare state typology. The results were almost unchanged after adjusting for sociodemographic factors, employment, and household characteristics. Nordic countries had the most favorable profile, followed by Anglo-Saxon countries, while Eastern and Southern European countries had the greatest financial strain. Differences between country typologies were more pronounced among women. On the other hand, gender differences within each country typology were much smaller than those between welfare state typologies.

**Table 2:** Association between financial strain and country group by gender.

	**Men**	**Women**								
	**%**	**cOR**	**95% CI**	**aOR**	**95% CI**	**%**	**cOR**	**95% CI**	**aOR**	**95% CI**
**Nordic**	**12.1**	**1**		**1**		**12.3**	**1**		**1**	
**Continental**	**27.6**	**2.76**	**2.15–3.54^a^**	**2.78**	**2.15–3.59^a^**	**31.0**	**3.21**	**2.49–4.15^a^**	**3.12**	**2.39–4.08^a^**
**Anglo-Saxon**	**17.8**	**1.57**	**1.20–2.05^b^**	**1.55**	**1.17–2.05^b^**	**24.4**	**2.30**	**1.76–3.02^b^**	**2.72**	**2.05–3.62^a^**
**Eastern European**	**41.4**	**5.12**	**3.98–6.60^a^**	**4.84**	**3.71–6.31^a^**	**44.3**	**5.67**	**4.38–7.36^a^**	**6.26**	**4.76–8.23^a^**
**Southern European**	**49.5**	**7.11**	**5.52–9.14^a^**	**5.84**	**4.49–7.61^a^**	**47.9**	**6.58**	**5.08–8.54^a^**	**6.16**	**4.69–8.10^a^**

*Crude (cOR) and adjusted odds ratios (aOR) and 95% confidence intervals (95% CI). 6th European Working Conditions Survey, 2015*.

*% refers to the prevalence of financial strain in each category*.

a *P < 0.05;*

b *P < 0.01. Odds ratios are adjusted for sociodemographic factors, employment, and household characteristics*.

### Financial Strain and Health Status

Table 3 shows the association between financial strain and health status. Among men, and for all welfare state typologies, financial strain was related to poor self-perceived health status and poor psychological well-being, although in Nordic countries these differences were not statistically significant for poor health status. The same pattern was observed among women, although the differences were not statistically significant for self-perceived health status in Anglo-Saxon countries, or for psychological well-being in Nordic countries. In both sexes, and for the two health indicators, the magnitude of the association was generally smaller in Southern European countries.

**Table 3:** Association between family financial strain and health by welfare state typology and gender.

**Men**															
	**Continental** ***N*** **= 5,142**			**Anglo-Saxon** ***N*** **= 1,995**			**Eastern Europe** ***N*** **= 2,592**			**Southern Europe** ***N*** **= 2,800**			**Nordic** ***N*** **= 627**		
	**%**	**aOR**	**95% CI**	**%**	**aOR**	**95% CI**	**%**	**aOR**	**95% CI**	**%**	**aOR**	**95% CI**	**%**	**aOR**	**95% CI**
**Poor self-perceived health status**															
No financial strain	10.3	1		11.6	1		13.8	1		16.8	1		13.2	1	
Financial strain	19.1	2.22	1.82–2.70^c^	19.8	1.79	1.27–2.52^b^	24.3	1.80	1.42–2.28^c^	22.8	1.28	1.02–1.60^a^	18.7	1.28	0.64–2.54
**Poor psychological well-being**															
No financial strain	7.1	1		17.8	1		10.9	1		8.9	1		10.4	1	
Financial strain	19.3	3.05	2.49–3.74^c^	25.1	1.57	1.17–2.11^b^	18.6	1.77	1.38–2.26^c^	14.9	1.85	1.41–2.41^c^	24.0	3.56	1.84–6.88^c^
**Women**															
	**Continental** ***N*** **= 5,109**			**Anglo-Saxon** ***N*** **= 1,919**			**Eastern Europe** ***N*** **= 2,871**			**Southern Europe** ***N*** **= 2,746**			**Nordic** ***N*** **= 580**		
	%	aOR	**95% CI**	**%**	**aOR**	**95% CI**	**%**	**aOR**	**95% CI**	**%**	**aOR**	**95% CI**	**%**	**aOR**	**95% CI**
**Poor self-perceived health status**															
No financial strain	9.0	1		7.6	1		13.6	1		19.2	1		7.9	1	
Financial strain	21.8	2.97	2.45–3.61^c^	9.6	1.38	0.91–2.10	24.2	1.86	1.51–2.31^c^	21.6	1.24	1.00–1.53^a^	17.1	2.61	1.19–5.72^a^
**Poor psychological well-being**															
No financial strain	10.5	1		19.5	1		10.5	1		10.4	1		13.0	1	
Financial strain	23.5	2.75	2.30–3.28^c^	31.7	2.56	1.94–3.37^c^	21.1	2.01	1.60–2.51^a^	17.7	1.74	1.37–2.22^c^	18.3	1.36	0.66–2.79

*Adjusted odds ratios (aOR) and 95% confidence intervals (95% CI). 6th European Working Conditions Survey, 2015*.

*Note: % refers to the prevalence of in each category*.

a *P < 0.05;*

b *P < 0.01;*

c *P < 0.001; odds ratios are adjusted for sociodemographic factors, employment and household characteristics*.

## Discussion

This study has produced three main findings: (1) there were large differences in the prevalence of financial strain between welfare state typologies, which were not explained by sociodemographic factors, employment status, or household characteristics; (2) differences across country typologies were more pronounced among women; and (3) in both sexes and in all welfare state typologies, financial strain was associated with poor self-perceived health status and poor psychological well-being although the magnitude of the association differed by welfare state typologies and gender.

### Prevalence of Financial Strain

After adjusting for sociodemographic, employment and household characteristics, there were large differences in the prevalence of financial strain between welfare state typologies and they were greater among women.

As expected, the prevalence of financial strain was lower in Nordic countries, which are characterized by high levels of both decommodification and defamilisation. However, Anglo-Saxon countries, ranked second, although they have the lowest levels of decommodification of all typologies examined, and also have low levels of defamilisation. It should be noted that the UK Working Tax Credit is the most important measure of its kind in Europe, both in terms of scope and budget (22). For working households receiving tax credits there is a reduction in the poverty gap of two-thirds. The Anglo-Saxon approach to reducing poverty emphasizes labor activation. In the UK, more than half of people who experienced in-work poverty in 1 year had exited by the following year, mostly by remaining in work but exiting poverty (38). The rather low levels of financial strain in Continental countries are likely due to their strongly regulated labor markets, together with traditional state economic support for families, which assumes that women have most responsibility for care at home (39). Eastern, and especially Southern European countries, where almost half of workers suffer from financial strain, had the highest prevalence of financial strain, which is consistent with previous studies (40). Both country typologies are characterized by low levels of defamilisation and, additionally, they have the highest labor market precariousness and lowest incomes (23, 41).

Differences in the prevalence of financial strain across welfare state regimes were larger among women which may be explained by a greater effect of differences in defamilisation levels among them. However, this is a speculation which deserves further research.

### Financial Strain and Health Status

Financial strain was related to poor self-perceived health status and poor psychological well-being in both sexes and in all country typologies, even after adjusting for age, immigrant status, occupational, and household characteristics. This finding is consistent with those of other studies, which report that current financial strain is strongly and independently associated with depression, even more than other traditional measures of socio-economic position (27, 42). However, it should be noted that in both sexes the magnitude of the association was generally smaller in Southern European countries. This finding is consistent with previous comparative studies reporting the greater health resilience of Mediterranean countries which could be related to a several factors (11) such as family solidarity, social support or diet, among others. A study carried out among African American families with incomes below 250% of the federal poverty level also found a positive relationship between social support and resilience to urban poverty (43). Studies carried out in Europe have shown that the magnitudes of health inequalities vary by welfare state regime but that this variation is not always in the direction expected as the Scandinavian countries do not exhibit the smallest health inequalities (44, 45).

### Strengths and Limitations

To our knowledge, this is the first study about financial strain and health status in a large and representative sample of employees from the EU28 countries. Unlike other studies based on the EU-SILC, in which only the head of the household was asked about financial strain, this study included all workers. The perceptions of the head household may differ from those of other household members (41). In contrast to many previous studies, we assessed and identified gender differences in the prevalence of financial strain across welfare state typologies.

The study also has some limitations. It has a cross-sectional study, so we cannot entirely rule out the possibility that poor health causes financial strain. However, we reduced this potential reverse causation bias by excluding individuals who reported LLI in the previous 6 months. Therefore, we have focused on a relatively healthy and homogeneous population and adjusted for a broad range of individual factors of financial strain and still we have found a consistent relationship between financial strain and health status.

## Conclusion

There are large differences in the prevalence of financial strain between welfare state typologies, which are independent of individual factors and more pronounced among women. Whereas, the levels of decommodification and defamilisation can explain to a great extent these cross-country differences, they are not correlated to the differences in the magnitude of the associations between financial strain and health status. Among employees of the EU28, financial strain is related to poor health status and poor psychological well-being in both sexes and in all welfare state typologies, independently of age, immigrant status, employment, and household characteristics. However, whereas Southern European countries have the highest prevalence of financial strain, the magnitude of the association with health status was smaller than in other country typologies.

The differences between welfare state typologies in the prevalence of financial strain, independently of its individual determinants as well as in the magnitude of the association between financial strain and health, highlight the need for policies that consider specific structural measures to reduce financial strain among workers.

## Data Availability Statement

Publicly available datasets were analyzed in this study. This data can be found here: Data come from the 6th European working conditions survey (https://www.eurofound.europa.eu/surveys/european-working-conditions-surveys/sixth-european-working-conditions-survey-2015).

## Author Contributions

LA and IC-F contributed in the conceptualization and design of the study. LA performed the statistical analyses, wrote the first draft of the paper, and confirms that all listed authors meet authorship criteria and that no others meeting the criteria were omitted. All the authors contributed in the interpretation and the discussion of the results, critically revised the manuscript, and approved the final version.

## Conflict of Interest

The authors declare that the research was conducted in the absence of any commercial or financial relationships that could be construed as a potential conflict of interest.
